# Inhaled [D-Ala^2^]-Dynorphin 1-6 Prevents Hyperacetylation and Release of High Mobility Group Box 1 in a Mouse Model of Acute Lung Injury

**DOI:** 10.1155/2021/4414544

**Published:** 2021-09-27

**Authors:** Vladislav N. Karkischenko, Veronika I. Skvortsova, Melik T. Gasanov, Yuriy V. Fokin, Maxim S. Nesterov, Nataliya V. Petrova, Oxana V. Alimkina, Igor A. Pomytkin

**Affiliations:** ^1^Scientific Center of Biomedical Technologies of the Federal Medical and Biological Agency of Russia, 143442 Svetlye Gory Village 1, Krasnogorsk District, Moscow Region, Russia; ^2^Faculty of Medical Biology, Pirogov Russian National Research Medical University, Russian Federation, Ostrovityanova Str. 1, 117997 Moscow, Moscow, Russia

## Abstract

COVID-19 is a respiratory infection caused by the SARS-CoV-2 virus that can rapidly escalate to life-threatening pneumonia and acute respiratory distress syndrome (ARDS). Recently, extracellular high mobility group box 1 (HMGB1) has been identified as an essential component of cytokine storms that occur with COVID-19; HMGB1 levels correlate significantly with disease severity. Thus, the modulation of HMGB1 release may be vital for treating COVID-19. HMGB1 is a ubiquitous nuclear DNA-binding protein whose biological function depends on posttranslational modifications, its redox state, and its cellular localization. The acetylation of HMGB1 is a prerequisite for its translocation from the nucleus to the cytoplasm and then to the extracellular milieu. When released, HMGB1 acts as a proinflammatory cytokine that binds primarily to toll-like receptor 4 (TLR4) and RAGE, thereby stimulating immune cells, endothelial cells, and airway epithelial cells to produce cytokines, chemokines, and other inflammatory mediators. In this study, we demonstrate that inhaled [D-Ala^2^]-dynorphin 1-6 (leytragin), a peptide agonist of *δ*-opioid receptors, significantly inhibits HMGB1 secretion in mice with lipopolysaccharide- (LPS-) induced acute lung injury. The mechanism of action involves preventing HMGB1's hyperacetylation at critical lysine residues within nuclear localization sites, as well as promoting the expression of sirtuin 1 (SIRT1), an enzyme known to deacetylate HMGB1. Leytragin's effects are mediated by opioid receptors, since naloxone, an antagonist of opioid receptors, abrogates the leytragin effect on SIRT1 expression. Overall, our results identify leytragin as a promising therapeutic agent for the treatment of pulmonary inflammation associated with HMGB1 release. In a broader context, we demonstrate that the opioidergic system in the lungs may represent a promising target for the treatment of inflammatory lung diseases.

## 1. Introduction

Coronavirus disease 2019 (COVID-19) is a respiratory infection caused by the SARS-CoV-2 virus. It can rapidly escalate to life-threatening pneumonia and acute respiratory distress syndrome (ARDS) [1]. It is generally accepted that a SARS-CoV-2-evoked elevation of cytokine levels above the normal range—commonly defined as a “cytokine storm”—is implicated in the deterioration in COVID-19 patients [2]. Meta-analyses reveal associations between increased levels of cytokines (primarily interleukin-6 (IL-6)) in peripheral blood and higher disease severity and mortality in COVID-19 [3–5]. However, the reported levels of cytokines, including IL-6, in patients with COVID-19 were profoundly lower than those typically observed in non-COVID-19 ARDS cases and in sepsis, thus casting doubt on the role of cytokine storms in the progression to ARDS with COVID-19 [6]. Recently, extracellular high mobility group box 1 (HMGB1) was identified as a mediator of inflammation in COVID-19 [7–9]. The average levels of HMGB1 were approximately 5- and 26-fold higher than the normal range in the peripheral blood of patients with nonsevere and severe COVID-19, respectively [8], reaching levels typically observed with severe sepsis [10]. These HMGB1 levels correlate significantly with the severity of clinical manifestations, such as the risk for intensive care unit admission and death, the severity of acute lung injury (ALI) and acute respiratory distress syndrome (ARDS), the degree of organic impairment, and peak D-dimer levels [9]; this finding points to extracellular HMGB1 as a possible major driver of inflammation in COVID-19.

High mobility group box 1 (HMGB1) is a nuclear nonhistone DNA-binding protein whose biological function depends on posttranslational modifications, its redox state, its cellular localization, and binding partners [11, 12]. In a pioneering study by Wang et al. [10], extracellular HMGB1 was identified as a late mediator of endotoxin lethality in sepsis. The receptor-mediated release of HMGB1 from the nucleus to the cytoplasm and then to the extracellular milieu requires the hyperacetylation of conserved lysine residues within nuclear localization sequences 1 (NLS1) and 2 (NLS2) [13, 14]. This acetylation is reversible and dependent on a balance between acetylation and deacetylation reactions catalyzed by histone acetyltransferases (HATs) and histone deacetylases (HDACs), respectively [15]. Sirtuin 1 (SIRT1), a member of HDAC family, reportedly downregulates HMGB1 hyperacetylation and secretion under conditions of inflammation [16–18]. When released from cells to the intracellular milieu, HMGB1 acts as a potent mediator of inflammation, binding to toll-like receptor 4 (TLR4) and the receptor for advanced glycation end-products (RAGE), thereby stimulating immune cells, endothelial cells, and airway epithelial cells to produce cytokines, chemokines, and other inflammatory mediators [11].

The mechanism by which SARS-CoV-2 induces HMGB1 release in COVID-19 remains unknown, although new evidence suggests that toll-like receptor 4 (TLR4) signaling may play a role in this process. TLR4 does indeed mediate the SARS-CoV-2 inflammatory response [19–22]. The SARS-CoV-2 trimeric spike protein interacts with and activates TLR4, with an affinity similar to many virus-receptor interactions [19]. The SARS-CoV-2 spike protein S1 subunit induces proinflammatory responses via TLR4 signaling in a manner similar to the action of lipopolysaccharide (LPS), a classic agonist of TLR4 [20–22]. A link between LPS-induced TLR4 activation and HMGB1 release is well-established; it involves intermediary production of type I interferons, primarily IFN-*β* [23], which then induces hyperacetylation and the release of HMGB1 via activation of the Jak/STAT1 signaling pathway [13, 24]. Based on this, a model of LPS-induced acute lung injury (ALI) [25] appears to be relevant in recapitulating SARS-CoV-2-induced pulmonary inflammation.

Growing evidence suggests that peptide *δ*-opioid receptor (DOR) agonists can alleviate the inflammation caused by activation of TLR4 [26–28]. [D-Ala^2^, D-Leu^5^] enkephalin (DADLE), a synthetic peptide agonist of DOR, ameliorates ischemia-reperfusion brain injury by inhibiting the TLR4/nuclear factor kappa B (NF-*κ*B) signaling pathway [26]. DADLE reduced serum levels of HMGB1 and the production of proinflammatory cytokines in a rat model of sepsis induced by cecal ligation and puncture [27], in which the role of TLR4 was shown [29]. Nonpeptide DOR agonists, in a contrary, may even potentiate TLR4-mediated inflammation, depending on their chemical structures [30, 31].

In our previous studies, we have demonstrated that [D-Ala^2^]-dynorphin 1-6 (leytragin), a potent peptide agonist of DOR [32], significantly improves survival rates and inhibits the expression of proinflammatory cytokines (predominantly IL-6) in the lungs of mice with LPS-induced ARDS/ALI [28, 33] (Fig. S1; Supplementary materials). However, the improvement in survival with leytragin in our experiments cannot be attributed to the inhibition of IL-6 signaling, as we did not find any effect of tocilizumab, an IL-6 receptor antagonist, on survival in this model [34]. This suggests another molecular target of leytragin, related to mice lethality induced by hyperactivation of TLR4 signaling, must exist. We hypothesize that extracellular HMGB1 may be such a target of leytragin in LPS-induced ALI and ARDS.

In the present study, we aim to investigate the effects of leytragin on lung HMGB1 expression and release in an experimental mouse model of LPS-induced ALI. We focus on the mechanisms underlying HMGB1 release, including hyperacetylation. Furthermore, we seek to clarify the role of SIRT1 and opioid receptors in mediating the effects of leytragin.

## 2. Materials and Methods

### 2.1. Materials

RNA isolation kit “RNA-extran” was purchased from Sintol (Moscow, Russia); cDNA synthesis kit “REVERTA-L” was purchased from (AmpliSens, Moscow, Russia); leytragin was purchased from Bion (Obninsk, Russia); trypsin/Lys-C Kits was purchased from Promega (Madison, WC, USA); Protease Inhibitor Cocktail II (ab201116) was purchased from Abcam (Cambridge, MA, USA). Zoletil, Xyla, and Antisedan were obtained from Virbac (France), Interchemie (Netherlands), and Orion Pharma (Finland), respectively. All other reagents were obtained from Sigma-Aldrich (Merck, St. Louis, MO, USA).

### 2.2. Mice

C57Bl/6 mice were obtained from the Stolbovaya Biomed Center (Moscow region, Russia). Male mice, 10-12-weeks of age, were used in all the studies. Animals were housed in groups of five per cage under 12 h light/12 h dark cycle at a temperature of 20 ± 2°C and 60-70% humidity. Food and water were available ad libitum. All delivered mice were kept for 1 week as an acclimatization period prior to performing any experiments. Studies were carried out in accordance with the Directive of the Ministry of Health of Russia “On Approval of the Rules of Good Laboratory Practice” as well as the EU Directive 2010/63/EU on the protection of animals used for scientific purposes. All experiments were approved by Ethical Committee of Scientific Center of Biomedical Technologies of the FMBA of Russia. All efforts were undertaken to minimize the potential discomfort of experimental animals. Animal studies are reported in compliance with the ARRIVE guidelines [35].

### 2.3. Induction of Acute Lung Injury

*α*-Galactosyl ceramide (*α*-GalCer) [36] and lipopolysaccharide from E. coli 055:B5 (LPS) were purchased from Sigma-Aldrich (Merck, St. Louis, MO, USA) and used in the induction of ALI as described in detail in [25]. Briefly, 100 *μ*l of *α*-GalCer at a concentration of 10 *μ*g·ml^−1^ (50 *μ*g/kg) was administered into each mouse by inhalation. After 24 h, the same mice were anaesthetized with a mixture of Zoletil (80 mg/kg) and Xyla (4 mg/kg) in a volume of 0.1 ml per mouse delivered intraperitoneally and then received an intratracheal injection of 50 *μ*l of LPS at a concentration of 6 mg·ml^−1^ (15 mg/kg). After that, mice received a subcutaneous injection of Antisedan (3 mg/kg) to improve recovery from the anesthesia.

### 2.4. Treatment Design

**Figure 1:**
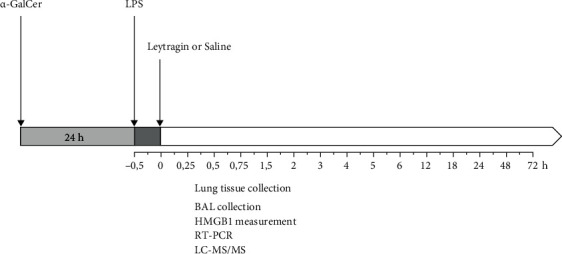
Experimental schedule of the present study.

Mice were randomized into groups, whereby the experimenter was blinded to treatment (for details on group size, see figure legends). Thirty minutes after the induction of ALI, the mice received once either 100 *μ*l of leytragin at a concentration of 0.02 *μ*g·*μ*l^−1^ (0.1 mg/kg of body weight) or 100 *μ*l of saline (control) by inhalation (Figure 1). The dose of 0.1 mg/kg was selected from our previous studies, as this dose reduced lethality in the mouse model of ALI [33]. In some experiments, naloxone was delivered intraperitoneally in a dose of 5 mg/kg just after the injection of LPS. Three to five mice from each group were anaesthetized with a mixture of Zoletil (80 mg/kg body weight) and Xyla (4 mg/kg body weight) and then euthanized by cervical dislocation at various time points within seventy-two hours after administration of leytragin or saline. In the first cohort of animals, bronchoalveolar lavage (BAL) samples were collected at 0.25, 0.75, 1.5, 3, 12, 18, 24, 48, and 72 hours for HMGB1 measurement. In the second cohort, BAL and lung tissue samples were collected at 0.25, 0.5, 0.75, 1, 1.5, 2, 3, 4, 5, 6, 12, 18, 24, 48, and 72 hours for all other analyses as described below.

### 2.5. BAL Collection

Samples of BAL were obtained as follows. After incision of the trachea, a plastic cannula was inserted, and airspaces were washed using 1.0 ml of saline (4 times with 0.25 ml). The BAL samples were then centrifuged (1000 × g for 5 min) at room temperature, and supernatants were collected. Protein content was quantified by Bradford assay. Samples were aliquoted and stored at −80°C until analyses.

### 2.6. Lung Tissue Collection

The lungs were excised, and lung samples of 100-150 mg wet weight were homogenized in 600 *μ*l of buffer consisting of Tris 0.5 M, NaCl 0.15 M, NP-40 0.5%, and Protease Inhibitor Cocktail II (ab201116) at 6500 rpm for 50 s using MagNa Lyser Rotor (Roche Diagnostics, Mannheim, Germany) followed by agitation at 120 rpm for 30 min. Then, the samples were centrifuged (5000 × g for 5 min) at room temperature, and supernatants were collected. Protein content was quantified by Bradford assay, and samples were aliquoted and stored at −80°C until analyses.

### 2.7. Measurement of HMGB1 Release

The concentrations of HMGB1 in BAL samples were measured using High Mobility Group Protein 1 ELISA kit (Cloud-Clone, Wuhan, China) according to the manufacturer's instructions and expressed in pg/ml.

### 2.8. Real-Time PCR

Total RNAs were extracted from the lung samples using the RNA-extran kit (Sintol, Moscow, Russia) and reverse-transcribed into complementary DNA using REVERTA-L kit (AmpliSens, Moscow, Russia) according to the manufacturer's instructions. RT-PCR reactions were performed on CFX96 Touch Real-Time PCR System (Bio-Rad laboratories, CA, USA). The mRNA expression of the target gene was calculated relative to *GAPDH*, a housekeeping gene. The sequences of oligonucleotide primers used in this study are shown in Table S1 (Supplementary materials). Results are presented as the mean fold change in mRNA expression of the target gene calculated by dividing the individual expression level in leytragin or saline treated mice by the average expression level of the naïve mice (*n* = 5).

### 2.9. Trypsin Digestion

Samples of BAL and lung tissues collected at 48 hours post leytragin or saline treatment as well as from naïve mice were subjected to trypsin digestion at 37°C for 18 hours using Trypsin/Lys-C kit (Promega, Madison, WC, USA) according the manufacturer's instructions. Each sample was centrifuged (5000 × g for 5 min) at room temperature and used for high-resolution liquid chromatography tandem mass-spectrometry (LC-MS/MS) analysis as detailed below.

### 2.10. Liquid Chromatography Tandem Mass Spectrometry Analyses

The tryptic peptides in each the sample (10 *μ*l) were separated by a linear gradient from 97% buffer A (0.1% formic acid in water)/3% buffer B (0.1% formic acid in acetonitrile) to 30% buffer A/90% buffer B within 45 min on Poroshell 120 EC-C18 column prior to MS detection using Agilent Technologies 1290 Infinity UHPLC, equipped with time-of-flight Agilent 6545 XT AdvanceBio LC/Q-TOF (Agilent Technologies, Santa Clara, CA, USA). Ionization was performed by electrospray (ESI) in a positive mode using a DuoJet Stream ESI source and an ion funnel. Analyses were performed in a full scan MS mode as well as in an automatic MS/MS mode in the mass range *m*/*z* 100-1700. To calibrate the internal mass throughout the analysis, purine ([M + H] + = 121.0509) and agilent compound HP0921 ([M + H] + = 922.0098) reference solutions were used. Data were processed using Agilent MassHunter Workstation software (Agilent B.08.01). All integrated peaks have been manually checked to ensure correct peak detection and accurate integration. The MassHunter BioConfirm program (Agilent B.09.00) was used to deduce the tryptic peptide sequence and establish its posttranslational modifications by searching the MS/MS spectra against protein sequence databases. Ratios of acetylated to nonacetylated (acetyl-K/K) peptides were calculated based on MS ion intensity (or peak area) for the selected tryptic peptides in a given sample.

### 2.11. Statistical Analysis

Data were analyzed using GraphPad Prism v.8.3.0 for Windows (San Diego, CA). Normality was tested with the Kolmogorov-Smirnov test. Statistical significance was determined by a one-way ANOVA with Tukey's post hoc test or two-way ANOVA followed by a Bonferroni's *post hoc* comparisons test. The level of confidence was set at 95% (*p* < 0.05).

## 3. Results

### 3.1. Leytragin Inhibits HMGB1 Release in LPS-Induced Mice

**Figure 2:**
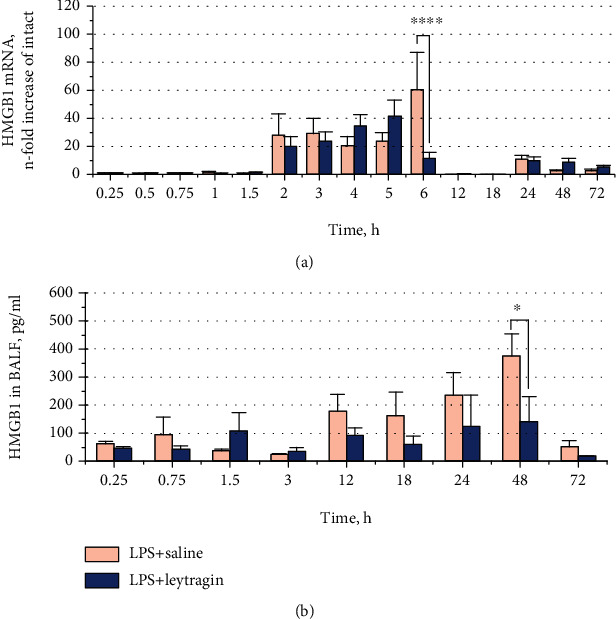
Effects of leytragin administration on LPS-induced elevations of HMGB1 release and mRNA levels. (a) Time courses for HMGB1 protein release into BAL in LPS-induced mice that received a single inhalation of leytragin or saline; 27–29 animals per group were used (3–4 per indicated time point). (b) The relative levels of HMGB1 mRNA in LPS-induced mice receiving inhalation of leytragin or saline; 75 animals per group were used (5 per indicated time point); ^∗^*p* < 0.05, ^∗∗∗∗^*p* < 0.0001 vs. LPS+saline, two-way ANOVA, and post hoc Bonferroni's test (see the text). Bars represent the mean ± SEM.

To test whether leytragin alters HMGB1 release, we measured concentrations of HMGB1 in the BAL of LPS-induced mice treated with leytragin or saline at various time points. A two-way ANOVA reveals significant main effects of leytragin (*F*_1,38_ = 5.640, *p* = 0.0227; Figure 2(a)) and time (*F*_8,38_ = 3.716, *p* = 0.0027) on HMGB1 concentrations in BAL of LPS-induced mice. Bonferroni's post hoc analysis shows that leytragin treatment significantly reduced the peak release of HMGB1 into BAL at the 48-hour time point (*p* < 0.05), compared to the control. Thus, leytragin treatment prevented HMGB1 release in the lungs of LPS-induced mice.

### 3.2. Leytragin Shows No Effect on HMGB1 mRNA Expression in the Lungs of LPS-Induced Mice

To investigate whether leytragin may affect LPS-induced HMGB1 mRNA expression, we compared the time courses of HMGB1 mRNA transcription in the lungs of LPS-induced mice treated with leytragin or saline for 72 hours. A two-way ANOVA revealed no significant main effect of leytragin administration (*F*_1,120_ = 0.3663, *p* = 0.5462; Figure 2(b)) on this transcription. Thus, administration of leytragin did not affect the LPS-induced elevations in HMGB1 mRNA expression.

### 3.3. Leytragin Prevents Hyperacetylation of HMGB1 in LPS-Induced Mice

**Figure 3:**
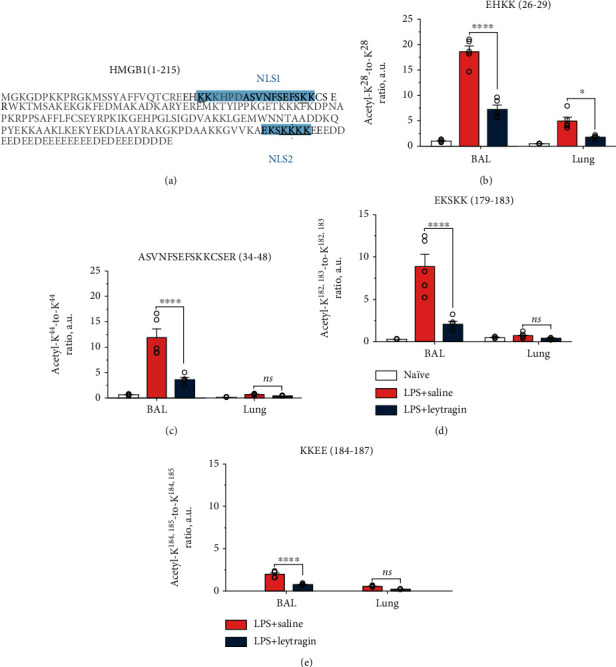
Effects of leytragin on the acetylation state of HMGB1 in samples of BALs and lungs from LPS-induced mice. (a) HMGB1 sequence with indicated tryptic peptides corresponding to HMGB1 fragments and overlapping with NLS sequences within HMGB1. Acetylated-to-nonacetylated ratios (acetyl-K/K) for (b) K^28^ within EHKK, (c) K^44^ within ASVNFSEFSKKCSER, (d) K^182,183^ within EKSKK, and (e) K^184,185^ within KKEE in BALs and lungs obtained from naïve mice and LPS-induced mice treated with leytragin or saline. Ns: nonsignificant, ^∗^*p* < 0.05, and ^∗∗∗∗^*p* < 0.0001 vs. LPS+saline. One-way ANOVA followed by Tukey's *post hoc* test (see the text); 5 animals per group were used. Bars represent the mean ± SEM.

To examine whether leytragin affects the LPS-induced hyperacetylation of HMGB1, we analyzed ratios of acetylated to nonacetylated tryptic peptides (acetyl-K/K) in samples of BAL and lungs after trypsin digestion, using LC-MS/MS (as described in Sections 2.8 and 2.9). Our analysis reveals the appearance of tryptic peptides corresponding to HMGB1 fragments 26–29 (EHKK), 34–48 (ASVNFSEFSKKCSER), 179–183 (EKSKK), and 184–187 (KKEE); these were acetylated at K^28^, K^44^, K^182,183^, and K^184,185^, respectively, where K^28^ and K^44^ belong to the NLS1 site while tandems K^182,183^ and K^184,185^ belong to the NLS2 site (Figure 3(a)). Notably, levels of acetylated peptides in samples from naïve mice were substantially lower than those from LPS-induced mice—and even undetectable in the case of the KKEE peptide. The numbering for amino acids follows the UniProt database (P63158) for mouse HMGB1. A one-way ANOVA reveals a significant difference between groups in acetyl-K/K ratios for K^28^ (*F*_5,24_ = 109.6, *p* < 0.0001; Figure 3(b)), K^44^ (*F*_5,24_ = 40.91, *p* < 0.0001; Figure 3(c)), K^182,183^ (*F*_5,24_ = 31.36, *p* < 0.0001; Figure 3(d)), and K^184,185^ (*F*_3,16_ = 71.07, *p* < 0.0001; Figure 3(e)). Tukey's post hoc tests reveal that LPS injection induced a significant increase in acetylation at K^28^, K^44^, and K^182,183^ in BAL samples (*p* < 0.0001) and at K^28^ in lung preparations (*p* < 0.001), compared to the respective naïve controls. *Post hoc* tests reveal also that treatment with leytragin significantly reduced LPS-induced acetylation at K^28^, K^44^, K^182,183^, and K^184,185^ in BAL samples (*p* < 0.0001) and at K^28^ in lung preparations (*p* = 0.02), but not at K^44^, K^182,183^, or K^184,185^ in lung preparations (*p* > 0.05), compared to the respective LPS-treated controls. Thus, the administration of leytragin prevented LPS-induced HMGB1 hyperacetylation at both NLS1 and NLS2 sites within HMGB1, with the most profound effect observed for HMGB1 molecules released to the BAL.

### 3.4. Leytragin Upregulates Sirtuin 1 mRNA Expression in LPS-Induced Mice

**Figure 4:**
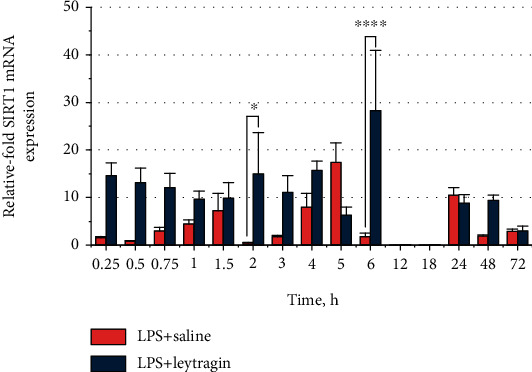
The treatment with leytragin increases SIRT1 mRNA levels in the lungs of LPS-induced mice. The relative levels of SIRT1 mRNA transcription in the lungs of mice that received leytragin were significantly higher than those in the control group; 75 animals per group were used (5 per indicated time point); ^∗^*p* < 0.05 and ^∗∗∗∗^*p* < 0.0001 vs. LPS+saline, two-way ANOVA, and *post hoc* Bonferroni's test (see the text). Bars represent the mean ± SEM.

To explore whether leytragin can affect SIRT1, we monitored the transcription of SIRT1 mRNA in the lungs of LPS-induced mice treated with inhaled leytragin or saline at various time points within 72 hours. A two-way ANOVA demonstrated significant main effects of leytragin (*F*_1,120_ = 26.32, *p* < 0.0001; Figure 4) and time (*F*_14,120_ = 2.971, *p* = 0.0006) on the transcription of SIRT1 mRNA in the LPS-induced mice. *Post hoc* analysis reveals that leytragin treatment significantly increased SIRT1 mRNA transcription at the six-hour time point (*p* < 0.0001; Bonferroni's test), compared to the saline group. Thus, administration of leytragin enhanced SIRT1 mRNA transcription in the lungs of LPS-induced mice.

### 3.5. Naloxone Abrogates the Effects of Leytragin on SIRT1 Expression in LPS-Induced Mice

**Figure 5:**
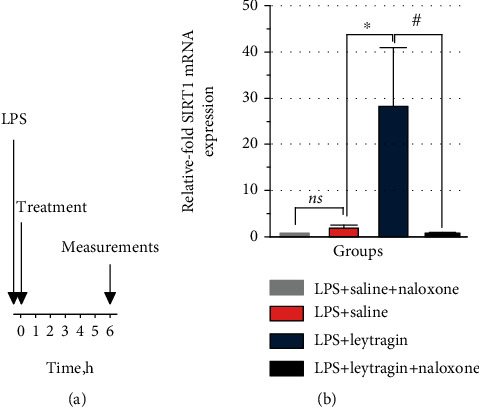
Naloxone abrogates the effects of leytragin on SIRT1 mRNA levels in LPS-induced mice. (a) Scheme of experiment. (b) The relative levels of SIRT1 mRNA transcription at the six-hour point after LPS installation; 5 animals per group were used; Ns: nonsignificant, ^∗^*p* < 0.05 vs. LPS+saline, and #*p* < 0.05 vs. LPS+leytragin+naloxone, one-way ANOVA followed by Tukey's *post hoc* test (see the text). Bars represent the mean ± SEM.

To clarify whether the effect of leytragin on SIRT1 expression is mediated through opioid receptors, we measured the expression of SIRT1 mRNA in the lungs of LPS-induced mice treated with inhaled leytragin or saline with or without naloxone, an antagonist of opioid receptors. A one-way ANOVA revealed a significant difference between the groups (*F*_3,16_ = 5.09, *p* = 0.012; Figure 5). Tukey's post hoc testing shows that the levels of SIRT1 mRNA in the lungs of LPS-induced mice treated by leytragin with naloxone were significantly higher (*p* < 0.05) than in those treated with leytragin alone. Thus, the effect of leytragin on the upregulation of SIRT1 mRNA transcription in LPS-induced mice is mediated by opioid receptors.

## 4. Discussion

The primary finding of our study is that leytragin, a peptide agonist of DOR, prevented HMGB1 release in the lungs of LPS-induced mice when administered by inhalation; this effect is mediated by opioid receptors, the upregulation of SIRT1 expression, and the downregulation of HMGB1 hyperacetylation at critical lysine residues within NLS1 and NLS2 sequences.

The hyperacetylation of HMGB1 is a crucial prerequisite for the active release of HMGB1 from cells into the extracellular environment [13, 14]. We demonstrate that leytragin inhibited the LPS-induced hyperacetylation of HMGB1 at both NLS1 and NLS2, with the greatest effect being observed for HMGB1 molecules released into BAL. Thus, the prevention of HMGB1 hyperacetylation appears to be the mechanism by which leytragin reduces HMGB1 release into the lungs of LPS-induced mice.

The acetylation state of HMGB1 is regulated by acetylation-deacetylation reactions catalyzed by histone acetyltransferases (HATs) and histone deacetylases (HDACs), respectively [15]. LPS shifts the balance toward HMGB1 hyperacetylation by triggering the degradation of HDAC4 via the TLR4/Jak/STAT1 signaling pathway [37]. SIRT1, another member of the HDAC family, is stable under conditions of inflammation and has been reported to downregulate LPS-induced HMGB1 hyperacetylation and release [16–18]. We have found that leytragin promoted SIRT1 mRNA transcription in the lungs of LPS-induced mice. We did not measure SIRT1 protein levels, since the SIRT1 protein undergoes rapid degradation in the fed state via the ubiquitin-proteasome pathway [38] (all animals in our study had free access to food over the 72-hour period of observation). Thus, the upregulation of *SIRT1* expression appears to be the mechanism by which leytragin shifts the acetylation-deacetylation balance toward HMGB1 deacetylation, thereby preventing HMGB1 release in LPS-induced mice.

DORs are the most prevalent type of opioid receptors in the lungs, particularly in the lung parenchyma [39]. We surmise that opioid receptors mediate the action of inhaled leytragin, since its effects on SIRT1 mRNA transcription in the lungs of LPS-induced mice were abrogated by naloxone, a nonselective antagonist of opioid receptors [40]. To our knowledge, this is the first example of an effect by a DOR agonist on SIRT1. Previously, morphine and fentanyl—nonpeptide agonists of *μ*-opioid receptors—have been reported to increase *SIRT1* expression in the mouse brain [41] and to inhibit NF-*κ*B in cancer cells in a SIRT1-dependent manner [42], respectively.

We have previously reported that inhaled leytragin inhibits the LPS-induced expression in the lungs of proinflammatory cytokines such as interleukin-1*β* (IL-*β*), tumor necrosis factor-*α* (TNF-*α*), and IL-6 [28], whose transcription is regulated by NF-*κ*B [43]. However, a mechanism underlying this effect of leytragin was then unknown. Our new finding that leytragin upregulates SIRT1 may explain the previous results, since SIRT1 is known to inhibit NF-*κ*B via the association with the RelA/p65 subunit of NF-*κ*B and because it deacetylates RelA/p65 on lysine 310 [44], a site that is critical for NF-*κ*B transcriptional activity [45, 46].

**Figure 6:**
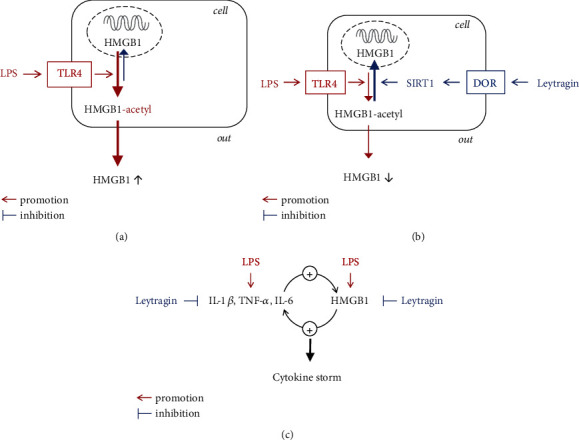
Schematic diagram illustrating the modes of leytragin action in a mouse model of LPS-induced pulmonary inflammation. (a) LPS via TLR4 triggers HMGB1 hyperacetylation, followed by HMGB1's translocation from the nucleus to the cytoplasm and then to the extracellular milieu [13, 24]. (b) Leytragin via DOR upregulates *SIRT1* expression, causing deacetylation of HMGB1 [16–18], thereby redirecting HMGB1 to the nucleus and preventing its release into the extracellular milieu. (c) Leytragin inhibits the LPS-induced production of proinflammatory cytokines, as well as HMGB1 release, thereby inhibiting the progression to cytokine storms and lethal ARDS.

Based on our results from previous research [28, 33] and this study, we draw several tentative conclusions regarding the mode of action of inhaled leytragin (Figure 6). A primary feature of leytragin is that it affects numerous targets involved in LPS-induced pulmonary inflammation and cytokine storms. Inhaled leytragin inhibits the lung expression of proinflammatory cytokines such as IL-*β*, TNF-*α*, and IL-6 (Fig. S2 and Fig. S3 and Table S2; Supplementary materials). Furthermore, leytragin inhibits the LPS-induced hyperacetylation of HMGB1 via upregulation of SIRT1 in an opioid receptor-dependent manner, thereby preventing HMGB1's release into BAL. Given that extracellular HMGB1 and these cytokines mutually amplify each other's production and release [10, 47], the multitarget action of leytragin appears to represent a promising way to alleviate cytokine storms, which involve elevated levels of both cytokines and HMGB1.

## 5. Conclusions

In this study, we provide evidence that leytragin, the peptide agonist of DOR, prevents the TLR4-mediated release of HMGB1 in the lungs. Given the role of the TLR4/HMGB1 axis in the progression of pulmonary inflammation—including with cytokine storms occurring with COVID-19—our results indicate that leytragin is a promising therapeutic agent for the prevention of cytokine storm with COVID-19. In a broader context, the opioidergic system in the lungs may present a promising target for the treatment of cytokine storms occurring with inflammatory lung diseases.

## Data Availability

The datasets used and/or analyzed during the current study are available from the corresponding author on reasonable request.

## Supplementary Materials

Supplementary Materials Table S1: oligonucleotide primers and PCR probes used in the study. Table S2: two-way ANOVA analysis of leytragin effects on mRNA levels of cytokines in the lungs of LPS-induced mice. Fig. S1: Kaplan-Meier survival curves (adapted from Karkischenko et al. [33] with permission from Journal Biomed). Fig. S2: cytokine storm in the lungs of LPS-induced mice. Fig. S3: effects of leytragin on mRNA levels of cytokines in the lungs of LPS-induced mice.
