# Supplementary material for: Inhaled [D-Ala2]-Dynorphin 1-6 Prevents Hyperacetylation and Release of High Mobility Group Box 1 in a Mouse Model of Acute Lung Injury
Source: J Immunol Res. 2021 Sep 27;2021:4414544. doi: 10.1155/2021/4414544 (PMC8490075; doi:10.1155/2021/4414544)
Supplement: Supplementary Materials — Table S1: oligonucleotide primers and PCR probes used in the study. Table S2: two-way ANOVA analysis of leytragin effects on mRNA levels of cytokines in the lungs of LPS-induced mice. Fig. S1: Kaplan-Meier survival curves (adapted from Karkischenko et al. [33] with permission from Journal Biomed). Fig. S2: cytokine storm in the lungs of LPS-induced mice. Fig. S3: effects of leytragin on mRNA levels of cytokines in the lungs of LPS-induced mice. [file 4414544.f1.pdf]

Inhaled [D-Ala^2]-dynorphin 1-6 Prevents Hyperacetylation and Release of High Mobility Group Box 1 in a Mouse Model of Acute Lung Injury

Vladislav N. Karkischenko[1], Veronika I. Skvortsova[2], Melik T. Gasanov[1], Yuriy V. Fokin[1], Maxim S. Nesterov[1], Nataliya V. Petrova[1], Oxana V. Alimkina[1] and Igor A. Pomytkin[1]*.

[1]Scientific Center of Biomedical Technologies of the Federal Medical and Biological Agency of Russia, 143442 Svetlye gory village 1, Krasnogorsk district, Moscow region, Russian Federation.
[2] Faculty of Medical Biology, Pirogov Russian National Research Medical University, Moscow, Russian Federation, Ostrovityanova str. 1, 117997 Moscow, Russian Federation

*Corresponding author: E-mail: ipomytkin@mail.ru

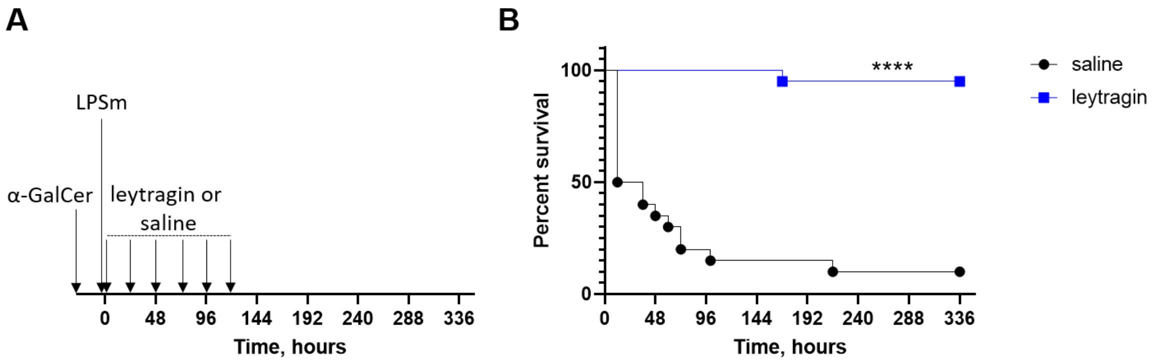

Supplementary Figure S1. Kaplan-Meier survival curves. Adapted from Karkischenko et al. [32] with permission from Journal Biomed (Russia). (A) Experimental schedule. C57Bl/6 mice received 50 µg/kg of α-galactosyl ceramide (α-GalCer) by inhalation and after 24 hours the same mice received a single intratracheal injection of a mixture (LPSm) consisting of lipopolysaccharide E. coli (LPS) 15 mg/kg, muramyl dipeptide 5 mg/kg, and 10 µl/mouse of Freund's complete adjuvant. 30 minutes after the LPSm injection, the mice received once-a-day for six consecutive days either leytragin 0.1 mg/kg by inhalation and 0.01 mg/kg i.m. or saline i.m. (control). (B) Survival curves for mice treated with leytragin or saline after LPSm challenge. 20 animals per group were used; $^{****}p < 0.0001$ vs. saline, Log-rank Mantel-Cox test.

Gene	Oligonucleotide primers and PCR probes
HMGB1	forward: 5′-GGG TTG TAA ATT GGC ATG GA-3′<br>reverse: 5′-GTC AAC AAA ACA GCC GCA AT-3′<br>probe: ROX-AGT TAT ATA TGG GGA CAG TAG TTT G-BHQ2
SIRT1	forward: 5′-TCC TTG GAG ACT GCG ATG TT-3′<br>reverse: 5′-ATG AAG AGG TGT TGG TGG CA-3′<br>probe: ROX-TGA GTT GTG TCA TAG GCT AGG TGG T-BHQ2
IL1B	forward: 5’-GAG AAC CAA GCA ACG ACA AA-3’<br>reverse: 5’-CTT GTT GAA GAC AAA CCG TT-3’<br>probe: ROX-TAA TGA AAG ACG GCA CAC CCA CCC T-BHQ2
IL6	forward: 5’-ATG AAG TTC CTC TCT GCA AG-3’<br>reverse: 5’-GTG TAA TTA AGC CTC CGA CT-3’<br>probe: ROX-CTT CTT GGG ACT GAT GCT GGT GAC A-BHQ-2
TNFA	forward: 5’-TCT GTC TCT CAC CTG CTC TG-3’<br>reverse: 5’-GGT TCT CAG ATG TGT CAC GA-3’<br>probe: ROX-GAA TGG ATG GGC TAC ATA AGT TAC G-BHQ2
IFNA	forward: 5’-ATC AAA CAG CCC AGA AGA CC-3’<br>reverse: 5’-GGC TTT CTT GTT CCT GAG GT-3’<br>probe: ROX-GGC TCT GTG CTT TCC TGA TGG TTT T-BHQ2
IFNB	forward: 5’-CAC CAC AGC CCT CTC CAT CA-3’<br>reverse: 5’-GCA TCT TCT CCG TCA TCT CC-3’<br>probe: ROX-GGC TCT GTG CTT TCC TGA TGG TTT T-BHQ2
VEGF	forward: 5’-GCA ACA GGC TGG ATG GGT AG-3’<br>reverse: 5’-CTC AGG TCA CTC CAA CTC CC -3’<br>probe: ROX-GCA TTT AGG GGG TCA GGG TGA AGC T-BHQ2

Supplementary Table S1. Oligonucleotide primers and PCR probes used in the study.

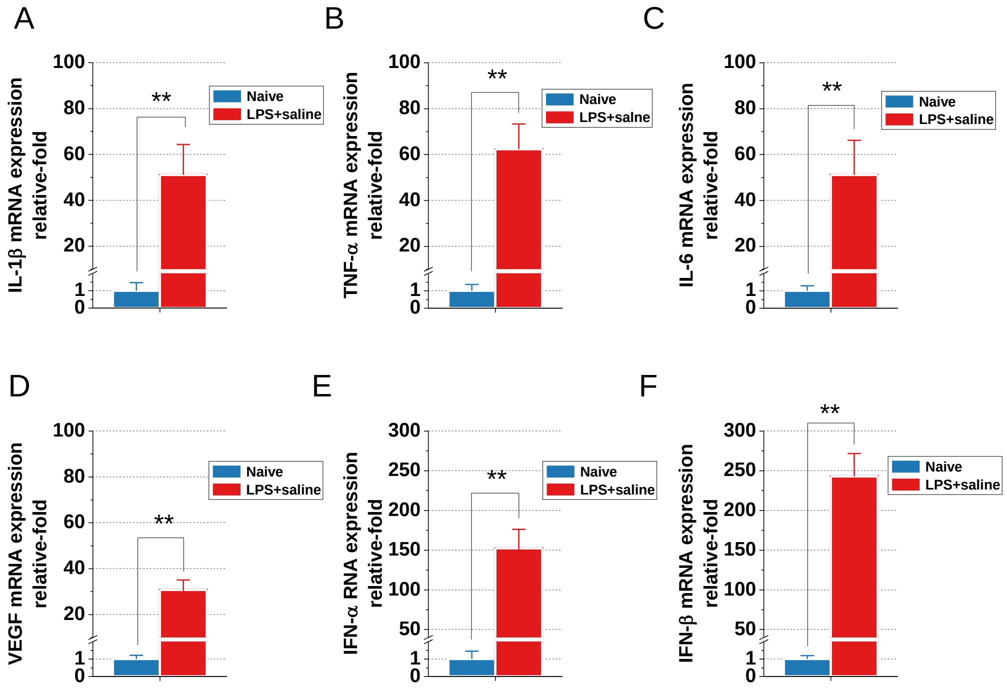

Supplementary Figure S2. Cytokine storm in the lungs of LPS-induced mice. The relative levels of interleukin-1β (IL-1β) (A), tumor necrosis factor-α (TNF-α) (B), interleukin-6 (IL-6) (C), vascular endothelial growth factor (VEGF) (D), interferon-α (IFN-α) (E), and interferon-β (IFN-β) (F) mRNA transcription in the lungs of naïve mice and LPS-induced mice at time point of 5.5 hours post LPS administration. 5 animals per group; $^{**}p < 0.01$, Mann–Whitney U-test. Bars represent Mean ± SEM relative-fold mRNA expression of the target gene calculated by dividing the individual expression level in naïve mouse or LPS-induced mouse by the average expression level of the naïve mice. Experiments were carried out as described in the "Materials and Methods" section.

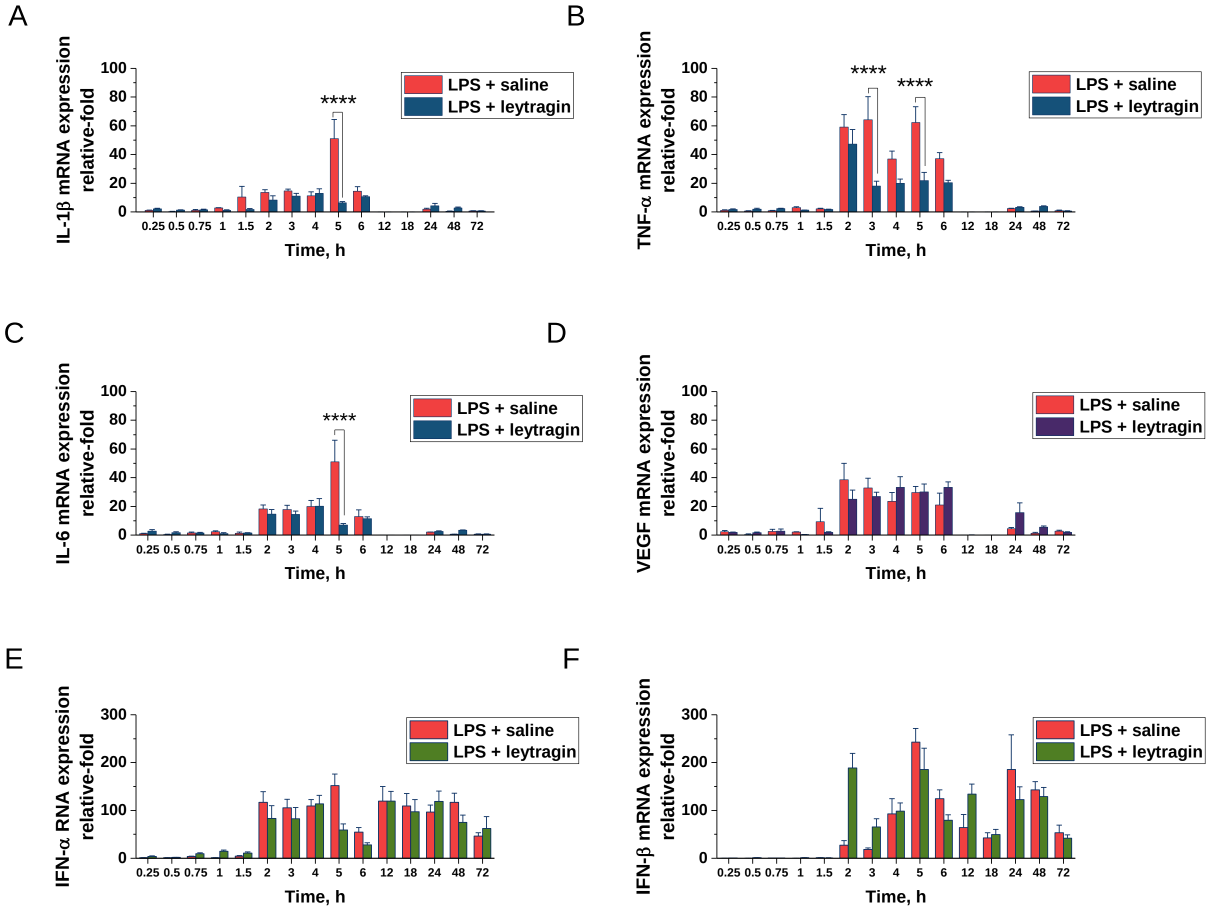

Supplementary Figure S3. Effects of leytragin on mRNA levels of cytokines in the lungs of LPS-induced mice. The relative levels of interleukin-1β (IL-1β) (A), tumor necrosis factor-α (TNF-α) (B), interleukin-6 (IL-6) (C), endothelial growth factor (VEGF) (D), interferon-α (IFN-α) (E), and interferon-β (IFN-β) (F) mRNA transcription in the lungs of LPS-induced mice that received leytragin or saline (control). 75 animals per group were used (5 per indicated time point); $^{****}p < 0.0001$ vs. LPS + saline, two-way ANOVA and *post-hoc* Bonferroni's test (see the Supplementary Table S2). Bars represent Mean ± SEM fold change in mRNA expression of the target gene calculated by dividing the individual expression level in leytragin or saline treated mice by the average expression level of the naïve mice (n = 5). Experiments were carried out as described in the "Materials and Methods" section.

Cytokine	Comparison	F (DFn, DFd)	P value	Significant
IL-1β	Time*Treatment	F (14, 120) = 7.249	<0.0001	Yes
	Time	F (14, 120) = 13.28	<0.0001	Yes
	Treatment	F (1, 120) = 12.30	0.0006	Yes
TNF-α	Time*Treatment	F (14, 120) = 5.453	<0.0001	Yes
	Time	F (14, 120) = 32.47	<0.0001	Yes
	Treatment	F (1, 120) = 24.00	<0.0001	Yes
IL-6	Time*Treatment	F (14, 120) = 5.930	<0.0001	Yes
	Time	F (14, 120) = 15.27	<0.0001	Yes
	Treatment	F (1, 120) = 6.688	0.0109	Yes
VEGF	Time*Treatment	F (14, 120) = 1.215	0.2732	No
	Time	F (14, 120) = 17.86	<0.0001	Yes
	Treatment	F (1, 120) = 0.1398	0.7091	No
IFN-α	Time*Treatment	F (14, 120) = 1.651	0.0751	No
	Time	F (14, 120) = 17.09	<0.0001	Yes
	Treatment	F (1, 120) = 3.171	0.0775	No
IFN-β	Time*Treatment	F (14, 120) = 3.192	0.0003	Yes
	Time	F (14, 120) = 18.81	<0.0001	Yes
	Treatment	F (1, 120) = 0.7197	0.3979	No

Supplementary Table S2. Two-way ANOVA analysis of leytragin effects on mRNA levels of cytokines in the lungs of LPS-induced mice. The main effects of time and treatment and their interactions were analyzed. There were 15 levels for time factor (0.25; 0.5; 0.75; 1; 1.5; 2; 3; 4; 5; 6; 12; 18; 24; 48; and 72 hours). There were 2 levels for treatment factor (LPS + saline, LPS + leytragin). 75 animals per group were used (5 per indicated time point).
